# Linguistic Laws in Speech: The Case of Catalan and Spanish

**DOI:** 10.3390/e21121153

**Published:** 2019-11-26

**Authors:** Antoni Hernández-Fernández, Iván G. Torre, Juan-María Garrido, Lucas Lacasa

**Affiliations:** 1 Societat Catalana de Tecnologia, Secció de Ciències i Tecnologia, Institut d’Estudis Catalans, Carrer del Carme 47, 08001 Barcelona, Catalonia, Spain; 2 Complexity and Quantitative Linguistics Lab, LARCA Research Group, Institut de Ciències de l’Educació, Universitat Politècnica de Catalunya, Av. Doctor Marañón 44-50, 08028 Barcelona, Catalonia, Spain; 3 Departamento de Matemática Aplicada y Estadistíca, Universidad Politécnica de Madrid, Plaza Cardenal Cisneros, 28040 Madrid, Spain; ivan.gonzalez.torre@upm.es; 4 Laboratorio de Fonética Antonio Quilis, Facultad de Filología, Universidad Nacional de Educación a Distancia, 28040 Madrid, Spain; jmgarrido@flog.uned.es; 5 School of Mathematical Sciences, Queen Mary University of London, Mile End Road, London E1 4NS, UK

**Keywords:** Zipf’s law, Brevity law, Menzerath–Altmann’s law, Herdan’s law, lognormal distribution, size-rank law, quantitative linguistics, Glissando corpus, scaling, speech

## Abstract

In this work we consider Glissando Corpus—an oral corpus of Catalan and Spanish—and empirically analyze the presence of the four classical linguistic laws (Zipf’s law, Herdan’s law, Brevity law, and Menzerath–Altmann’s law) in oral communication, and further complement this with the analysis of two recently formulated laws: lognormality law and size-rank law. By aligning the acoustic signal of speech production with the speech transcriptions, we are able to measure and compare the agreement of each of these laws when measured in both physical and symbolic units. Our results show that these six laws are recovered in both languages but considerably more emphatically so when these are examined in physical units, hence reinforcing the so-called ‘physical hypothesis’ according to which linguistic laws might indeed have a physical origin and the patterns recovered in written texts would, therefore, be just a byproduct of the regularities already present in the acoustic signals of oral communication.

## 1. Introduction

Linguistic laws are statistical regularities and properties of linguistic elements (i.e., phonemes, syllables, words or sentences) which can be formulated mathematically and estimated quantitatively [1]. While linguistic laws have been thoroughly studied over the last century [1,2,3], the debate on its ultimate origin is still open. In what follows we summarise the classic four linguistic laws [3], recently revised, mathematically substantiated and expanded to two other laws [4] (see Table 1 for specific formulations):

1. Zipf’s law. After some notable precursors (as Pareto [5], Estoup [6] or Condon [7] among others), George Kingsley Zipf formulated and explained in [8,9] one of the most popular quantitative linguistic observations known in his honor as Zipf’s Law. He observed that the number of occurrences of words with a given rank can be expressed as $f\left(r\right)∼r^{-\alpha }$, when ordering the words of written corpus in decreasing order by their frequency. This is a solid linguistic law proven in many written corpus [10] and in speech [11], even though its variations have been discussed in many contexts [12,13,14].
2. Herdan’s law. Although with little-known precedents [15], Herdan’s law [16] (also known as Heap’s law, because it was also formulated later by Heaps in [17]) describes that the average growth of new different words *V* in a text of size *L* follows $V∼L^{\alpha },\alpha <1$ [16]. Thus, Herdan’s law shows the evolution of the number *V* of different words in a text (types) as its size increases, measured in the total number of words (*L*). *L* obviously is obtained by the summation of the number of occurrences of each word (tokens), for each different words types that appear in the text.
3. Brevity law. Also known as Zipf’s law of abbreviation, its original qualitative statement claims that the more a word is used, the shorter it tends to be [8,9,18]. In texts or transcriptions, usually the way of measuring the word size is using the number of characters that compose the word. In this way, brevity law has been empirically proven in texts from almost a thousand languages of eighty different linguistic families [19], but also holds acoustically when measuring the time duration of words [29,30].
  The leap from the classical qualitative conception of brevity law to a quantitative proposal has recently been made [4,31]. In information-theoretic terms [32], if a certain symbol *i* has a probability $p_{i}$ of appearing in a given symbolic code with a D-ary alphabet, then its minimum (optimal) expected description length $\ell_{i}^{*}=-\log_{D}\left(p_{i}\right)$. Deviating from optimality can be effectively modelled by adding a pre-factor, such that the description length of symbol *i* is $\ell_{i}∼-\frac{1}{\lambda_{D}}\log_{D}\left(p_{i}\right)$, where $0<\lambda_{D}\leq 1$. So, the closer $\lambda_{D}$ is to one, the closer it is the system to optimal compression. Reordering terms, one finds an exponentially decaying dependence between the frequency of a unit and its size (see [4] for further details on the mathematical formulation).
4. Size-rank law. Zipf’s law and brevity law involve frequencies. Taking advantage of the new mathematical formulation of the latter, these can now be combined [4] in such a way the “size” $\ell_{i}$ of a unit *i* is mathematically related to its rank $r_{i}$ via α (Zipf) and λ (brevity law) exponents. Experimentally, $\theta =\frac{\alpha }{\lambda }$ is therefore an observable parameter which indeed combines Zipf and Brevity exponents in a size-rank plot, and this law predicts that the larger linguistic units tend to have a higher rank following a specific logarithmic relation [4].
5. Menzerath-Altmann law. Again after some forerunners [20], Paul Menzerath established that there is a negative correlation between the length of a linguistic construct and the length of its constituents [21,22]. Subsequently, a mathematical formulation law was heuristically proposed by Gabriel Altmann [23,24]: if *n* stands for the size of the linguistic construct and *y* is the constituent size, then $y\left(n\right)=an^{b}\exp\left(-cn\right)$, being *a*, *b* and *c* free parameters of the model, whose interpretation remains controversial [33]. Definitely, Menzerath–Altmann’s law could be simplified and generalized qualitatively as “the longer a language construct the shorter its components (constituents).” [23,34]. This law has been revised in different linguistic levels under multiple and polyhedral perspectives [1,33,34], but above all in written texts. Recently some researchers are turning back to the phonetic origins of the law [35] and new mathematical models explaining the actual formulation have been proposed [4].
6. Lognormality law. Previous studies have found consistently lognormal distributions for spoken phonemes in several languages [25,26,27,28,36] and in word and breath groups (BGs) duration for English [4,37]. In [4] it was confirmed that the time duration of phonemes, words and breath groups in speech are well described by lognormal distribution for the English language. Moreover, in [4] a general stochastic model was presented to explain and justify such lognormality at all linguistic levels only assuming that the lowest (phonemic) level follows a lognormal distribution, hence claiming the universal validity of the lognormal shape and its proposal as a ‘lognormality law’.

A critical review of the literature about linguistic laws shows that the majority of empirical studies have been conducted by analyzing written corpus or transcripts, while research on linguistic laws in speech have been limited to small and fragmented corpora. In this work, we have followed the protocol of [4] to systematically explore linguistic laws in speech in two new languages: Catalan and Spanish. We simultaneously use both physical and symbolic magnitudes to examine the abovementioned laws at three different linguistic levels in physical and symbolic space. We certify that these hold approximately well in both languages, but better so in physical space. As we will see, this new evidence in Catalan and Spanish further support the physical hypothesis as an alternative to the classical approach of the so-called symbolic hypothesis in the study of language. On one hand, the symbolic hypothesis states that these statistical laws emerge in language use as a consequence of its symbolic representation [4]. On the other hand, the physical hypothesis claims that the linguistic laws emerge initially in oral communication—possibly as a consequence of physical, acoustic, physiological mechanisms taking place and driven by communication optimization pressures—and the emergence of similar laws in written texts can thus be regarded as a byproduct of such more fundamental regularities [4,38,39]. We aim to recover in this way a more naturalistic approach to the study of language, somehow cornered after many years of written corpus studies in computational linguistics.

## 2. Results

Our results are based on an analysis of Glissando corpus [40]. For illustration, Table 2 summarises some general characteristics of this oral corpus (for more details, see Section 4, Materials and Methods). For the phonetic inventory of Spanish and Catalan, note that only phonemes that appear effectively in the Glissando corpus have been taken into account, without considering other phonemes that could appear in other linguistic varieties of both languages [41].

In the next subsections we provide a systematic study of each of the six laws detailed above in Glissando corpus. Table 3 summarizes the fitted exponents and parameters for all the linguistic laws explored in this work at the level of words, whereas Table 4 does the same at the phonemic level, with the exception, as explained in the following section, of the lognormality law for Catalan and Spanish. As we will see, linguistic laws are again recovered with only slight differences with respect to English [4] and some technical details that are worth detailing for each law.

### 2.1. Lognormality Law and Low-Resolution Effects

**Figure 1:**
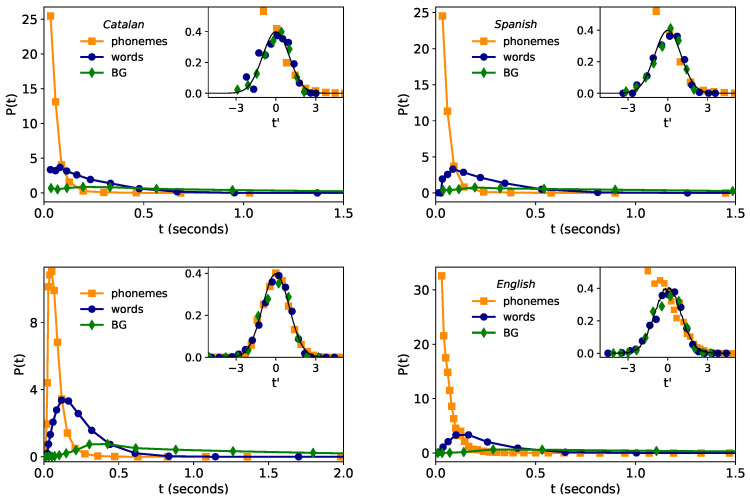
Lognormality law for time duration. (**outer panels**) Time duration distribution of phonemes (orange), words (blue) and BGs (green) for Glissando corpus: Catalan (**top left**) and Spanish (**top right**). For comparison, in the bottom left panel we show the results of English from Buckeye corpus (extracted from [4]), where Buckeye has finer statistics (higher resolution) than Glissando. A coarsened version of the English corpus—developed to be comparable with Glissando’s resolution—is plotted in the bottom, right panel (see the text for details). (**inset panels**) Collapse of all distributions after time rescaling $t^{\prime }=\left(\log\left(t\right)-\langle \log\left(t\right)\rangle /std\left(\log\left(t\right)\right)\right)$ (where std(log(t)) stands for the standard deviation of the random variable logt). If time durations at all levels comply with a lognormal distribution, then the collapsed data should approach a standard Gaussian N(0,1) (solid line), in good agreement with the results. Small deviations found in Catalan and Spanish are similarly found in the coarsened version of English, thus concluding that such deviations are mainly due to finite-precision and lower-bound detectability effects, and the lognormality law otherwise holds.

Recently [4], after exploring most common plausible family of probability distributions with the use of maximum likelihood estimation method (MLE) [44], compelling statistical evidence showed that the time duration distribution in speech in an English corpus is lognormally distributed across linguistic scales (phonemes, words, and BG), and such regularity was robust for individual speakers. Moreover, a generative mechanism able to explain the stability of the lognormal law for different linguistic scales was also proposed [4], suggesting that such regularity is indeed universal, hence proposing the so-called lognormality law. Here we explore the fulfillment of such new law in two additional languages, Catalan and Spanish. Empirical results for the time duration distribution P(t) of phonemes, words, and breath-groups (BGs) are depicted in the main plots of the top panels of Figure 1. Since a lognormal distribution appears normal (Gaussian) in linear-logarithmic axis, we have logarithmically rescaled the time duration variable *t* as

$$t^{\prime }=\frac{\log(t)-\langle \log(t)\rangle }{\sigma (\log(t))}.$$

Accordingly, if P(t) is lognormal, then $P(t^{\prime })$ is a standard Gaussian N(0,1) regardless of the linguistic scale. This fact is numerically checked in the inset panels of Figure 1.

Overall the data approximately collapse to the Gaussian shape—hence validating the lognormality law—however, there are small deviations, and these are notably stronger for phonemes at short timescales (note that only the right branch of the Gaussian is recovered and deviations are found at $t^{\prime }<0$). In what follows we argue that this is indeed an artifact due to finite-precision and lower-bound resolution of the Catalan and Spanish corpus, rather than a genuine, linguistic effect.

First, note that lower-bound segmentation time in the Glissando Corpus is 30 ms, and the corpus has a precision (granularity) of 10 ms. The lower-bound segmentation time precludes us from experimentally observing the left-end of the phoneme time duration distribution. Furthermore, these artifacts can propagate up to a higher scale (i.e., to words), as evidenced by the fact that words with duration of 30, 40 or 50 ms turn out to be always composed by a single phoneme, words with time duration of 60, 70 and 80 ms have one or two phonemes, and so on.

In order to certify that these low-resolution issues are indeed underpinning the deviations from the pure lognormality law, we have added the following experiment. The so-called Buckeye corpus (English corpus) has higher resolution than Glissando and precision and is, therefore, free from these issues (also, Buckeye corpus has larger sample sizes than Glissando, see Table 2). Indeed for the Buckeye corpus, compliance to the lognormality law has recently found to be excellent (see bottom left panel of Figure 1). We thus proceed to construct a coarsened, low-resolution version of the Buckeye corpus, comparable to the particularities of the Glissando corpus under study, by rounding up time durations in Buckeye data to a precision of 10 ms and, by further setting the minimum observable time duration (the lower limit segmentation) to 30 ms (we do not deal with further limitations such as that words shorter than 60 ms are always composed of one phoneme in Glissando). The resulting time distribution of phonemes, words, and BGs in this coarsened Buckeyed corpus are plotted in the right panel of Figure 1. Interestingly, similar deviations from the lognormality law to the ones found in the Glissando corpus are now recovered in the low-resolution version of the Buckeye corpus. This evidence supports our hypothesis that the lognormality law indeed holds well in Catalan and Spanish, albeit it might not be fully observable at the phoneme level in the Glissando corpus. Furthermore, this analysis flags an important issue: low-resolution effects such as low precision and a too-large lower limit segmentation time can induce important deviations and hinder the observation of the true, underlying distribution.

**Figure 2:**
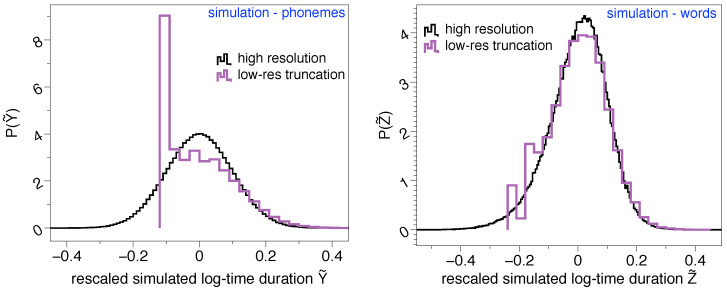
Lognormality law truncation. (**left**) Rescaled log-time duration distribution of synthetic ‘phonemes’ $P(\tilde{Y})$, estimated by (i) sampling Y=exp(X) where *X* is normally distributed $X∼(\mu ,\sigma^{2})$ with μ=−3, σ=2, and then (ii) rescaling $\tilde{Y}=\left[\log Y-\langle \log Y\rangle \right]/std\left(\log Y\right)$ (where std(logY) stands for the standard deviation of the random variable logY). If *Y* is lognormal, then $\tilde{Y}∼N\left(0,1\right)$. (**right**) Rescaled log-time duration distribution of synthetic ‘words’ $P(\tilde{Z})$, obtained using the stochastic model of [4] by concatenating *n* phonemes where *n* is another random variable whose distributed is approximated empirically. As the left panel, if *Z* is lognormal, then $\tilde{Z}∼N\left(0,1\right)$. In both panels, the black curve is the original, high resolution experiment whereas the purple curve is the result of (i) reducing the precision by rounding off to two decimal digits, (ii) reducing the sampling size to match differences between Buckeye and Glissando, and (iii) impose a lower-bound detectability τ=0.03 s (akin to the 30 ms of Glissando), such that all synthetically generated phonemes with a duration Y<τ are rounded to 0.03 s. Whereas lognormality is recovered in the original experiment, this shape is smeared out as soon as the lower-bound detectability threshold and other low-resolution artifacts are imposed, thereby explaining why the lognormality law might not be fully observable in Glissando.

To further investigate these effects, it is worth discussing at this point that the origin of the lognormality law has been mathematically discussed recently in terms of a stochastic model [4]. Suppose that phoneme time durations can be modeled by a random variable *Y*, which is indeed lognormally distributed. Since words can be understood as concatenation of phonemes, then the time duration of words can thus be modeled by a random variable $Z=\sum_{i=1}^{n}Y_{i}$, where each $Y_{i}$ is in principle a different lognormal distribution and *n* is yet another random variable which describes the number of phonemes shaping up a word. Whereas when *n* is large the central limit theorem predicts *Z* is asymptotically normal when *n* is small and under some additional conditions, *Z* is well approximated by a lognormal distribution [4], thereby explaining why the time duration of words is indeed found to be lognormally distributed in practice. Now, how would *Z* be distributed if we imposed on its sampling the artifacts detected in Glissando, such as a large lower-bound detectability threshold, finite precision, or a smallish sample size? To illustrate these effects, we have run a numerical test where we initially sample words of duration *Z*, constructed by concatenating phonemes with time duration *Y* where Y=exp(X) and *X* is a Gaussian random variable. *Y* is therefore lognormal and if we log-rescale it $\tilde{Y}=\left[\log Y-\langle \log Y\rangle \right]/std\left(\log Y\right)$ (where std(logY) stands for the standard deviation of the random variable logY) we should recover a standard Gaussian N(0,1). This distribution is shown (black curve) in the left panel of Figure 2, whereas the case of words is plotted in the right panel of the same figure, approximately recovering again the lognormal shape (standard Gaussian in rescaled units). Then, we have repeated the same experiment and ‘lowered its resolution’ by imposing the following: (i) the precision of *Y* is rounded to two decimal digits, imitating the precision of 10 ms found in Glissando, (ii) any synthetic phoneme shorter than a lower bound Y<0.03 s is forced to have the minimal allowed duration, Y=0.03 s. Results for this low-resolution version of the original experiment are then shown as purple curves in the same Figure 2. In particular, we can see how the lognormal shape of the phoneme time duration is significantly affected for shorter timescales, and such issues propagate to the word case at short timescales. The phenomenology is similar to what we found by comparing the results on the Buckeye corpus (English) versus the same results on a low-resolution version of the Buckeye corpus (bottom panels of Figure 1). All in all, this provides yet additional evidence explaining why the lognormality law might not be fully observable across all linguistic scales if the corpus has these kinds of limitations.

### 2.2. Zipf’s Law for Words and Yule Distribution for Phonemes

**Figure 3:**
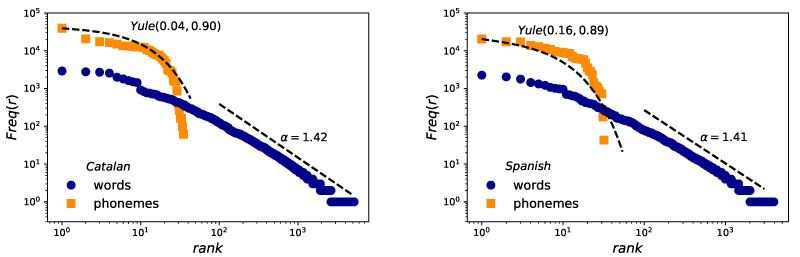
Zipf’s law. Log-log frequency-rank of phonemes (orange squares) and words (blue circles) for the case of Catalan (**left**) and Spanish (**right**). Words are fitted to a power law distribution following [45,46] and leading to $x_{min}=1$ and slopes almost similar for both languages. Phonemes are fitted to a Yule distribution with the help of the maximum likelihood estimation method (MLE).

Results for Zipf’s law are reported in Figure 3. The estimation of exponent α obtained for word frequencies applying the methodology of Clauset et al [45,46] are in agreement with those previously found [4] for the second regime in English (see Table 3), with α≈1.41 (Spanish and English) and α≈1.42 (Catalan), pointing to the robustness of the law also in speech. However, in the case of phonemes, whereas a Yule distribution can be fitted following the MLE method [44], fits are not very good—perhaps due to lack of statistics—and there are some slight differences between the distribution parameters of Catalan, Spanish and English (see Table 4). We conclude at this point that the Yule shape might not be universal for phoneme distribution and this hypothesis should be carefully revisited.

### 2.3. Herdan–Heaps’s Law

**Figure 4:**
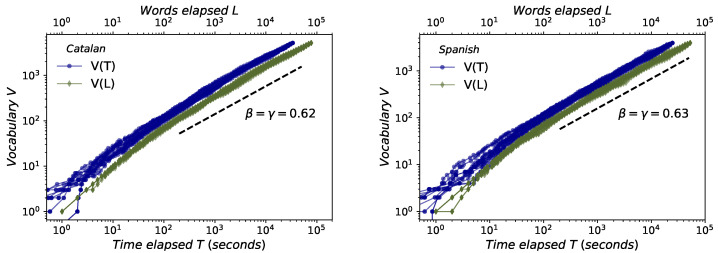
Herdan–Heaps’s law. Sublinear increase of number of different words *V* versus time elapsed *T* (blue circles) and versus total number of words spoken *L* (green diamonds) for Catalan (**left**) and Spanish (**right**). As we are leading with a multiauthor corpus, each line represents a different way of permuting the order of concatenating each speaker. In every case we find scaling laws $V\left(L\right)∼L^{\beta }$ and $V\left(T\right)∼T^{\gamma }$ which holds for about three decades. The scaling exponents β and γ are estimated for each permutation using the least-squares method, and the average value of each of them over all permutations is shown in the figure. We find β≈γ, as previously justified in [4], while its numerical value is on agreement with the one found for English [4].

This law accounts for the sublinear increase of the number of different words *V*, and can be measured in physical units (i.e., as a function of the time elapsed *T*, $V\left(T\right)∼T^{\gamma }$) or in symbolic units (i.e., as a function of the total number of words spoken *L*, $V\left(L\right)∼L^{\beta }$). Results are reported in Figure 4, certifying that (i) this law holds both in Catalan and Spanish and (ii) both in symbolic (β) and physical (γ) units, (iii) with a scaling exponent β≈γ, in good agreement to previous results [4] found for English: β≈0.63 (Spanish and English) and β≈0.62 (Catalan). In fact, this does not come as a surprise, given that a number of works have derived an inverse relationship between Zipf’s and Herdan’s exponents using different assumptions (see [47] or [48] for a review).

### 2.4. Brevity Law

**Figure 5:**
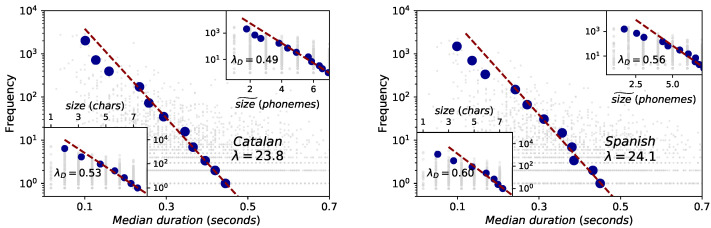
Brevity law: words (Catalan on the left panel and Spanish on the right panel). Red dashed lines are fits to the exponential law f∼exp(−λℓ), where *ℓ* is the word size which can be measured in physical units (mean duration) (**outer panels**) or in symbolical units (number of phonemes or number of characters, inset panels). See the text for and Table 3 for data fits and interpretation. Blue dots are the result of a data binning. Note that the fits are performed to the raw data, but the resulting exponential shape accurately matches the binned data within a range (deviations occur for shorter sizes, when the resolution and finite-precision issues of the Glissando corpus are important). Spearman test shows consistent negative correlations for the three formulations for the case of Catalan of −0.27, while for the case of Spanish the correlation is slightly stronger in physical magnitudes (−0.25) than in symbolic units (−0.22).

A mathematical formulation of brevity law was developed in [4] based on the information-theoretic principle of compression [32,49], when the size of the units is expressed in symbolic and physical units. In Figure 5 we report the results obtained for brevity law in the Glissando corpus for the case of words (left panel for Catalan, right panel for Spanish). Raw data (light grey) was fitted to the theoretical exponential law (see Table 1), and the best fit is depicted as a red dashed line. Also, a data binning is added (blue dots) to be able to visually compare it with the fit (red dashed line).

When word size is measured in physical units (word duration), the best exponential fit to the raw data (red dashed line) accurately matches the binned data, with similar fitting parameters λ≈23.8 (Catalan) and λ≈24.1 (Spanish) (to be compared with λ≈20.6 for English in Buckeye Corpus [4]), with significant Spearman correlations. Note that deviations of binned data from the red dashed line take place for short timescales: we argue that these are indeed related to the finite-precision and resolution issues discussed before, which propagate into (short) words.

When word size is measured in symbolic units (i.e., in the number of phonemes and number of characters), the law is again recovered (inset panels of Figure 5). Interestingly, the mathematical formulation of this law assigns a specific interpretation of the exponent λ when units are measured in symbolic space (i.e., when a code is available): the exponent in this case is always bounded 0≤λ≤1 and quantifies the deviation of the language under study from compression optimality, where the closer to 1 the closer to optimality [4]. Out of the three languages, results suggest that Spanish ($\lambda_{D}=0.56$ for phonemes and $\lambda_{D}=0.60$ for characters) is slightly closest to optimality, followed by English ($\lambda_{D}=0.5$ for phonemes and $\lambda_{D}=0.6$ for characters) and Catalan ($\lambda_{D}=0.49$ for phonemes and $\lambda_{D}=0.53$ for characters).

**Figure 6:**
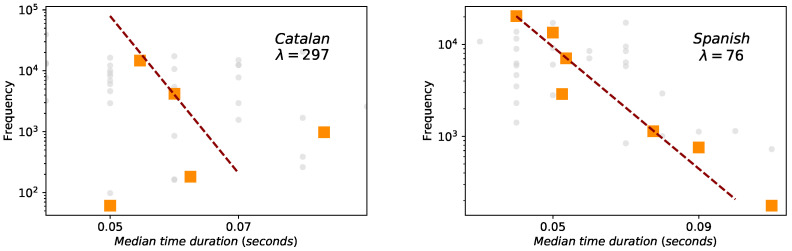
Brevity law: phonemes (Catalan on the left panel and Spanish on the right panel). Red dashed lines are fits to the exponential law f∼exp(−λℓ), where *ℓ* is the phoneme size measured in physical units (mean duration). Orange squares are the result of a data binning. Spearman test always denote negative correlations (−0.3 for Catalan, −0.54 for Spanish) but the data sample is too small to evaluate the agreement to the exponential law.

In the case of phoneme duration the statistics—and thus fit—are much poorer, especially for Catalan (we recall again on the finite-precision and resolution issues of Glissando corpus). Nevertheless, Spearman correlations are significant, both for Spanish and Catalan (Figure 6), although Spearman’s correlation is better for Spanish (−0.54) than for Catalan (−0.3).

### 2.5. Size-Rank Law

**Figure 7:**
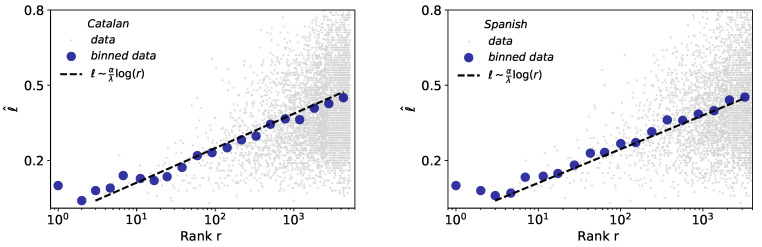
Size-rank law for words. Linear-log representation of word size *ℓ* versus rank of all words (blue dots denote binned data) in Catalan (**left**) and Spanish (**right**). The black dashed line is a fit of raw data (light grey dots) to the size-rank law (see Table 1), i.e., the fit of this law is not done to the binned data, however its agreement is excellent.

The size-rank law mathematically connects the Brevity and Zipf’s laws, indicating that the words of larger rank tend to have larger size [4]. Results for the case of words are depicted in Figure 7. Interestingly, despite the precision problems of Glissando for short durations already described previously, the size-rank law holds more robustly than the brevity law for both Catalan and Spanish. The slight variations in the exponent θ of Catalan (0.06) and Spanish (0.058), with respect to English (0.07), are here a consequence of the variations in the λ exponents of brevity law (Table 3).

### 2.6. Menzerath–Altmann’s Law (MAL)

**Figure 8:**
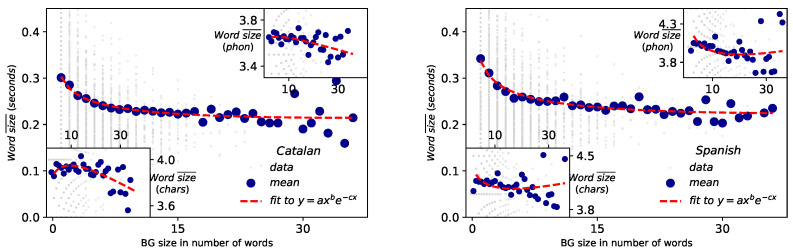
Menzerath–Altmann law: BG vs words Representation of BG size measured in number of words versus the mean size of those words for Catalan (**left**) and Spanish (**right**), where the size of the words can be measured in physical magnitudes (**main panel**) or symbolic units (phonemes or number of characters, inset panels). Each grey point represents one BG, whereas blue circles are the mean duration of BGs. MAL holds in physical magnitudes (with coefficient of determination $R^{2}=0.47$ for Catalan and $R^{2}=0.84$ for Spanish), while it is poorly fulfilled when the size is measured symbolically (Catalan: $R^{2}=0.23$ for character units and $R^{2}=0.11$ for phoneme units; Spanish: $R^{2}=0.04$ for character units and $R^{2}=0.08$ for phoneme units). Fitted parameters a,b,c are reported in Table 3.

**Figure 9:**
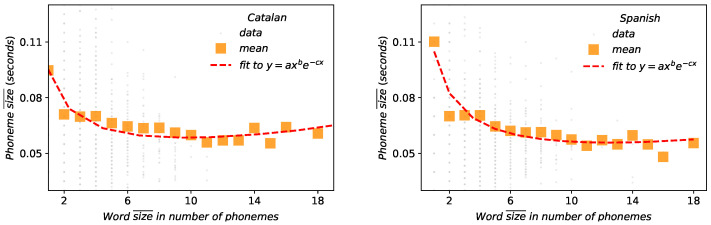
Menzerath–Altmann law: words–phonemes relation between the word size measured in number of phonemes versus the size of those phonemes in physical magnitudes. Orange squares represent the mean size of each word. Fitted parameters are shown in Table 4 coefficient of determination for these are $R^{2}=0.75$ for Catalan and $R^{2}=0.9$ for Spanish.

The results of fittings of the Catalan and English corpus to MAL for different scales are depicted in Figure 8 and Figure 9. For the scale of BGs vs words (Figure 8), MAL holds well when the size of the constituent is measured in physical units of time duration (outer panels) and it is either poorly or not fulfilled when the size is measured in symbolic units such as number of letters or number of phonemes per word (inset panels). Coefficients of determination $R^{2}=0.47$ for Catalan and $R^{2}=0.84$ for Spanish when size is measured in time duration, to bee compared with Catalan $R^{2}=0.23$ (Catalan, characters), $R^{2}=0.11$ (Catalan, phonemes), $R^{2}=0.04$ (Spanish, characters) and $R^{2}=0.08$ (Spanish, phonemes). These results are in agreement with the case of English [4]. Overall, better agreement to MAL is found for Spanish than for Catalan in time duration. Results and agreement to MAL also hold at the word vs phoneme scale. In fact, these results are new clear evidence in favor of the acoustical origin of the law [21] and the physical model explained in [4]. Note that while the size of the BGs are not large enough to reach to observe the range where MAL is inverted (at b/c≈34 words [4]), the value of the exponents (see Table 3 and Table 4) certifies that such regime inversion indeed exists.

## 3. Discussion

The relevance of the study of the frequency of linguistic elements and its relative marginalization by the academy in the twentieth century was eloquently highlighted by Bybee [50] (p.6), stating that:

The other major theoretical factor working against an interest in frequency of use in language is the distinction, traditionally traced back to Ferdinand de Sausurre (1916), between the knowledge that speakers have of the signs and structures of their language and the way language is used by actual speakers communicating with one another. American structuralists, including those of the generativist tradition, accept this distinction and assert furthermore that the only worthwhile object of study is the underlying knowledge of language (Chomsky 1965 and subsequent works). In this view, any focus on the frequency of use of the patterns or items of language is considered irrelevant.

While agreeing with Bybee [50], let us add here that the relevance of the study of the frequency of linguistic elements goes beyond the mere study of texts and, indeed, also concerns orality, which at the end of the day is at the basis of most human languages—with obviously some notable exceptions such as sign languages. In this work, we have studied the statistical properties and the onset of linguistic laws in the structure of speech in Catalan and Spanish. Set aside a precedent in pre-phonemic levels [39], to the best of our knowledge this is the first study of these characteristics in these two languages. Not only the frequencies of phonemes, words or breath groups of Catalan and Spanish have been examined here in speech transcriptions, but, perhaps more importantly, the presence of similar linguistic laws in these languages has been analyzed in inherently acoustic magnitudes (time duration), confirming some results previously found for English [4] and finding new evidence. Let us now summarise some of these findings and discuss some open problems for future research.

First, we have concluded that in order to fully observe the lognormality law at all linguistic scales the corpus under study needs to have sufficiently high resolution, and in particular a sufficiently low segmentation time, as close as possible to the physiological limit given by the glottal pulse (about 10 ms, see [51]). For too large lower-bounds (30 ms in the case of Glissando) and finite-precision, strong deviations from the lognormality law at the phoneme level will necessarily emerge, and some of these effects can mildly percolate at the word level. While it is not a definite proof, our experimental and numerical investigations supports our hypothesis that the lognormality law indeed holds well in Catalan and Spanish, complementing previous results in English [4], albeit it might not be fully observable at the phoneme level in Glissando corpus.

With respect to Zipf’s law for the frequency of words, as well as with Herdan’s law and the size-rank law, the experimental evidence found here for Catalan and Spanish reinforces the universality of these linguistic laws in speech. The mathematical models that relate these laws to each other are fully verified [4]. We can also highlight here the empirical strength of the size-rank law, very robust despite the variability of the data and the mentioned limitations of the corpus.

In the case of the brevity law for words, in Catalan and Spanish, the mathematical formulation derived from information theory and optimal coding [31] recently developed [4] is fully verified, and the law holds quantitatively. In a recent work [31] it has been established that optimal non-singular coding predicts that the length of linguistic elements should grow approximately as the logarithm of its frequency rank, which is consistent with Zipf’s law of abbreviation and our approach [4], but more work is needed to certify any optimality ranking between languages.Future work shall also extend this analysis to other languages (ideally from different linguistic families beyond the Indo–European) one and/or with different writing systems, and would also explore the connection between the brevity exponents and other complexity metrics of language or the so-called orthographic transparency, defined as the more or less direct relationship in the conversion between graphemes and phonemes for each language [52].

Finally, MAL has been fully certified to hold in Catalan and Spanish *only* when measured in physical units, in line with previous evidence for English [4] and providing additional evidence to suggest that this is indeed a fundamentally acoustic law. In a similar vein, the bulk of results provided in this work is yet another empirical support to the validity of the ‘physical hypothesis’, and we hope it provides encouragement for other researchers to follow-up and address the necessary challenges to fully verify this hypothesis and its implications for theoretical linguistics.

To round off let us discuss some additional open problems, potential objects of future research. In the case of the Spanish written corpus, the frequencies of the words, lemmas and even their punctuation marks have been extensively reviewed before [53], and similarly for the case of Catalan and Spanish—in a bilingual context—the evolution of words and lemmas during childhood and adolescence in written production has even been analyzed [54]. In future work, a similar approach could be explored within speech, by analyzing in detail whether linguistic regularities emerge both in pauses, interruptions and other elements of acoustic variability and also in prosody, which turn out to be fundamental for example in clinical linguistics [55]. Finally, in relation to the ontogeny of language, and similarly to recent works which study the evolution of Zipf’s law in language acquisition [13] as well as the law of brevity [56], the evolution of linguistic laws in speech is an open problem which also deserves investigation.

## 4. Materials and Methods

Glissando is a speech corpus for Spanish and Catalan which has been specially designed for prosodic studies, but that can be used also for other purposes [40]. It includes more than 12 hours of speech in Catalan and Spanish, recorded under optimal acoustic conditions, orthographically transcribed, phonetically aligned and annotated with prosodic information (location of the stressed syllables and prosodic phrasing). They are composed of three subcorpora: the ‘news’, a corpus of read news texts; the ‘Task dialogues’, a set of three task-oriented dialogues, covering three different interaction situations; and the ‘free dialogues’, a subcorpus of informal conversations. In this article, we aimed to research on orality and spontaneous conversation and compare the results with previous studies in English [4], so from those three we focused on ‘task dialogues’ and ‘free dialogues’.

We had simultaneous access to (i) the aligned speech signal, (ii) its symbolic transcription and (iii) its phonetic transcription. In this way, we had control over all linguistic levels of phonemes and words, while the annotations allowed us to define another linguistic level: the breath group (BG). BGs are defined as the sequence of utterances between pauses in speech for breathing or longer [57]. A more detailed description of the Glissando corpus can be found in [40].

Annotation files containing symbolic and phonetic transcription were derived from the orthographic transcription using an automatic phonetic transcription tool, a phonetic aligner and a tool for the automatic annotation of prosodic boundaries [58]. The phonetic transcription tool converted the orthographic text into a chain of phonetic symbols, representing the theoretical pronunciation of the text. This phonetic transcription was time-aligned with the speech signal using the phonetic aligner, which established where to locate the initial and final boundaries for each phoneme. These aligners have some margin of error in the placement of these boundaries, so the output of this automatic process is usually revised by hand [40]. In the case of the ‘News’ subcorpus, this automatic annotation was manually revised by several experts in Phonetics, to obtain a transcription of the actual pronunciation of the speakers and not a theoretical one, as provided by the automatic tools, and to correct misplaced boundaries. During this process, some phonetic symbols not necessarily related to Spanish or Catalan were included to transcribe anomalous or deviated pronunciations. The ’dialogues’ subcorpus, however, was not subject to this revision process [40]. This lack of manual revision might explain some of the specific phenomenology observed in this work, such as the lower-bound time duration resolution segmentation of the corpus, which could indeed be related to the limitations of the phonetic aligner.

### Data and Reproducibility

From the whole corpus, we have used data corresponding to ‘Free dialogues’ and ’Task dialogues’ in order to make the results more comparable with a previous work [4] which was based in the analysis of spontaneous speech conversation. Glissando corpus is a freely accessible corpus for non-commercial uses. It can be obtained through ELRA (http://catalog.elra.info/en-us/repository/browse/ELRA-S0406/ for the Spanish subcorpus and http://catalog.elra.info/en-us/repository/browse/ELRA-S0407/ for the Catalan subcorpus.).

Post-processed data of the Glissando corpus created by the authors of this article are available at https://doi.org/10.6071/M3XW9T, while scripts for generating the results are available at https://github.com/ivangtorre/ling-law-speech-spanish-catalan. We used Python 3.7 for the analysis. Levenberg–Marquardt algorithm, Kolmogorov–Smirnov distance, Spearman test and most of MLE fits use Scipy 1.3.0. MLE fits for power laws that are self-coded. Other libraries such as Numpy 1.16.2, Pandas 0.24.2 or Matplotlib 3.1.0 were also used. Fits to Zipf’s law are done with R and PowerRlaw [46].

## Figures and Tables

**Table 1:** **Main linguistic laws**, according to Torre and collaborators [4]. From left to right columns: name of the linguistic law, its mathematical formulation, details on its magnitudes and parameters; and finally some basic references about each law. While Zipf’s law is naturally defined and measured in symbolic units (texts or speech transcriptions), Herdan-Heaps, Brevity, Size-Rank and Menzerath-Altmann laws can be measured both in symbolic and physical units. Lognormality law is only defined in physical units (time duration).

	Mathematical Formulation	Details	References
Zipf’s law	$f\left(r\right)∼r^{-\alpha }$	*f*: frequency *r*: rank α: parameter	[5,6,7,8,9]
Herdan-Heaps’ law	$V∼L^{\beta }$	*L*: text size/time elapsed *V*: vocabulary β: parameter	[15,16,17]
Brevity law	f∼exp(−λℓ),λ>0	*f*: frequency *ℓ*: size λ: parameter	[4,8,9,18,19]
Size-rank law	$\ell ∼\theta \log\left(r\right),\;\theta =\frac{\alpha }{\lambda }$	*ℓ*: size *r*: rank θ: parameter	[4,9,18]
Menzerath-Altmann’s law	$y\left(n\right)=an^{b}\exp\left(-cn\right)$	*n*: size of the whole *y*: size of the parts a,b,c: parameters	[4,20,21,22,23,24]
Lognormality law	$p\left(t;\mu ,\sigma \right)=\frac{1}{t\sigma \sqrt{2\pi }}e^{-\frac{{\left(\ln\left(t\right)-\mu \right)}^{2}}{2\sigma^{2}}}$	*t*: time duration σ,μ: parameters	[4,25,26,27,28]

**Table 2:** Main characteristics of Glissando. This Table summarises main characteristics of Glissando corpus [40] across linguistic levels for both Catalan and Spanish. For reference, a comparison to Buckeye corpus (English) is provided [4,42,43]. We report the total number of linguistic elements (phonemes, words and breath groups (BG)), specifying the number of different linguistic elements (types) and the total (tokens). Since time duration distribution of linguistic levels are usually heavy-tailed [4], we use median duration (instead of mean) as a reference.

	Number of Elements					Median Duration (secs.)		
	Phonemes		Words		BG	Phon	Word	BG
	Tokens	Types	Tokens	Types	Tokens			
Catalan	$3\times 10^{5}$	35	$8\times 10^{4}$	$5\times 10^{3}$	$2\times 10^{4}$	0.05	0.20	0.8
Spanish	$2\times 10^{5}$	32	$5\times 10^{4}$	$4\times 10^{3}$	$1\times 10^{4}$	0.05	0.21	0.9
English	$8\times 10^{5}$	64	$3\times 10^{5}$	$9\times 10^{3}$	$5\times 10^{4}$	0.07	0.20	1.1

**Table 3:** Summary of exponents and parameters for the case of words. Results are on reasonable good agreement to those found for English in [4]. Note that actual fit of lognormality law in Spanish and Catalan was not carried out due to low-resolution problems of the Glissando corpus, however we certified that this law also holds (see the text).

Words	Zipf	Herdan-Heaps	Brevity	Size-Rank	Menzerath-Altmann			Lognormality	
	α	β	λ	θ	a	b	c	μ	σ
Catalan	1.42	0.62	23.8	0.060	0.301	−0.132	−0.004	-	-
Spanish	1.41	0.63	24.1	0.058	0.336	−0.148	−0.003	-	-
English	1.41	0.63	20.6	0.07	0.364	−0.227	−0.0067	−1.62	0.66

**Table 4:** Summary of exponents and parameters for the case of phonemes. Results are on reasonable good agreement to those found for English in [4]. Note that actual fit of lognormality law in Spanish and Catalan was not carried out due to low-resolution problems of the Glissando corpus, however we certified that this law also holds (see the text).

Phonemes	Yule		Brevity	Menzerath-Altmann			Lognormality	
	a	b	λ	a	b	c	μ	σ
Catalan	0.04	0.90	297	0.092	−0.355	−0.037	-	-
Spanish	0.16	0.89	76	0.102	−0.393	−0.032	-	-
English	0.25	0.96	127	0.18	−0.23	−0.007	−2.68	0.59

## Abbreviations

The following abbreviations are used in this manuscript:

BG	Breath group
LND	Lognormal distribution
MAL	Menzerath–Altmann’s Law
